# Association between childhood ADHD problems and premature mortality: identifying modifiable cardiovascular mechanisms in a UK population cohort

**DOI:** 10.3389/fpsyt.2026.1764335

**Published:** 2026-05-11

**Authors:** Ajay K. Thapar, Stephan Collishaw, George Davey Smith, Evie Stergiakouli, Anita Thapar

**Affiliations:** 1 Division of Psychological Medicine and Clinical Neurosciences, Centre for Neuropsychiatric Genetics and Genomics, Wolfson Centre for Young People’s Mental Health, Cardiff University School of Medicine, Cardiff, United Kingdom; 2 Population Health Sciences and Medical Research Council (MRC) Integrative Epidemiology Unit, University of Bristol, Bristol, United Kingdom

**Keywords:** ADHD, cardiovascular risk, childhood, mediation, mortality

## Abstract

**Background:**

Individuals with attention deficit hyperactivity disorder (ADHD) are at increased risk of premature mortality, but the mechanisms that underlie this association after young adulthood are unknown. As ADHD is associated with cardiovascular disease, modifiable cardiovascular risk factors could contribute to links between ADHD and premature mortality.

**Aims:**

This study aims to investigate whether specific cardiovascular risk factors explain the association between childhood ADHD problems and a higher risk of premature mortality.

**Methods:**

We used the UK 1958 birth cohort, the National Child Development study (NCDS), and linked death register data to examine whether children with ADHD problems at age 7 years were at higher risk of premature mortality by age 58 and if specific modifiable cardiovascular risk factors, measured at midlife (age 44 years), mediated this association using path analysis.

**Results:**

A total of 8,016 individuals completed both the age 7 ADHD assessment and the age 44/45 biomedical assessment. Of these individuals, 231 (3.1%) were grouped as likely having ADHD. The odds ratio (OR) for deaths (*n* = 251) in the ADHD group versus the non-ADHD group was 1.86 (95% CI 1.08–3.17). The risk was largely explained by cigarette smoking status at midlife and by a higher waist–hip ratio (a measure of obesity).

**Conclusions:**

Childhood ADHD problems are associated with a higher risk of premature mortality by age 58. This risk seems to be mainly explained by two potentially modifiable cardiovascular risk factors: obesity and smoking. These risks should be prioritized for preventative interventions to reduce the risk of premature mortality in those with a history of ADHD.

## Introduction

It is well established now that individuals with attention deficit hyperactivity disorder (ADHD) are at increased risk for premature death, and this includes natural causes (1–8). In fact, a recent UK paper based on primary care data found that individuals with ADHD were living 7–9 years less than those without ADHD (8). Although these findings are concerning, the mechanisms that explain the risk for premature mortality are unknown. ADHD is known to be associated with a wide variety of physical health conditions, some of which are life-threatening, so ill-health is one possible explanation. ADHD has consistently been found to be associated with cardiovascular disease (9, 10), with Mendelian randomization suggesting that this link may be causal (11, 12). As cardiovascular disease is the leading cause of mortality in the UK and many of its risk factors such as hypertension, hyperlipidemia, and obesity are modifiable, we investigated whether specific cardiovascular risk factors explain the association between childhood ADHD problems and increased premature mortality rate in a UK-population-based cohort.

## Materials and methods

### Sample

The National Child Development Study (NCDS) is a prospective UK birth cohort comprising 18,558 children born in England, Wales, and Scotland on March 3–9, 1958 and followed up by questionnaire at regular intervals every few years, with an additional face-to face biomedical assessment at 44 to 45 years of age with a research nurse, which included a detailed assessment of their physical health. A comprehensive description of the NCDS study has been published (13).

All procedures involving human subjects/patients were approved by South East MREC (NCDS Biomedical, Age 44, 2002, REC number 01/1/44).

### Measures

#### Exposure: ADHD group

At the age of 7 years, the mothers of the participants in the NCDS were asked to complete a modified parent-rated Rutter A scale which is a questionnaire measure of different child mental health and neurodevelopmental problems, including ADHD, and has been validated for the diagnosis of ADHD (14). The items included whether the child “is squirmy or fidgety” or “has difficulty in settling.” The response categories were “never”, “sometimes”, and “frequently”. Teachers were also asked about two ADHD symptoms in the participants (“squirmy, fidgety” and “hardly ever still”) at similar time points. The two teacher items were derived from the Bristol Social Adjustment Scale (BSAG) (16) with response categories “don’t know”, “certainly applies”, “applies somewhat”, and “does not apply”. Individuals have previously been classified as having broadly defined childhood ADHD if the parent endorsed the child, at age 7 years, as having both ADHD symptoms (regardless of frequency) and the teacher endorsed either of the ADHD symptoms as “certainly applies”. A clinical diagnosis of ADHD requires difficulties across different settings. Broadly defined ADHD shows association with the same risk factors, correlates, and outcomes as a strict diagnosis (15).

#### Outcome: mortality by age 58 years

This was derived from the National Child Development Study Deaths Dataset, 1958–2016. This listed the month and year of death of any cohort member (up until 2016). We recoded this into a binary variable: alive or dead post-1965 (i.e., the age at which ADHD was assessed).

#### Mediators of risk: cardiovascular risk factors

Obesity and waist hip ratio (WHR): Waist and hip circumference and height and weight were measured by nurses during home visits at age 44 years. BMI was derived using the standard formula (BMI = weight (kg)/height (m^2^)). All of those with a BMI ≥30 kg/m were classified as obese. Additionally, in line with research indicating that waist–hip ratio (WHR) may be a better indicator of obesity than BMI in terms of cardiovascular-related mortality (16), we also derived the WHR (waist circumference (cm)/hip circumference (cm)).

Current smoker: This was a derived dichotomized item (current smoker/current non-smoker) based on self-reported responses to a question, at age 42 years, asking about what would best describe their smoking status (non-smoker/ex-smoker/smoke occasionally/smoke daily).

Hazardous alcohol use: We used a well-established method to assess this—an AUDIT (17) score ≥8. This was based on responses to the AUDIT questions incorporated in the biomedical interview at ages 44–45 years.

Systolic blood pressure (mmHg): Three readings of systolic (SBP) and three readings of diastolic (DBP) blood pressure were available. A mean value for each (SBP and DBP, in mmHg) was derived. Given previous evidence, we only considered systolic blood pressure as the primary CVD risk factor (18).

Lipid measurements (mmol/L): Low-density lipoprotein (LDL) and triglyceride (TG) level data were also available.

### Analysis

#### Bivariate associations

Initially, we carried out bivariate analysis (linear or logistic regression as appropriate to outcome) to examine associations between (1) ADHD and mortality status (whether alive or dead by age 58) and (2) individual cardiovascular risk factors and mortality status. The relationship between ADHD problems and cardiovascular risk factors in NCDS has been reported in a previous paper (Thapar et.al., 2022). However, given that the focus here is on premature mortality, we included two additional cardiovascular risk factors: (1) hazardous alcohol intake (AUDIT score ≥8) and (2) waist–hip ratio (as an index of obesity). As we had planned to test for these associations, all testing was based on existing empirical evidence, and only a relatively limited number of tests were carried out. We did not correct for multiple testing. The analyses were conducted using SPSS Version 27.

#### Identifying the mediators of the association between ADHD and premature mortality

**Figure 1:**
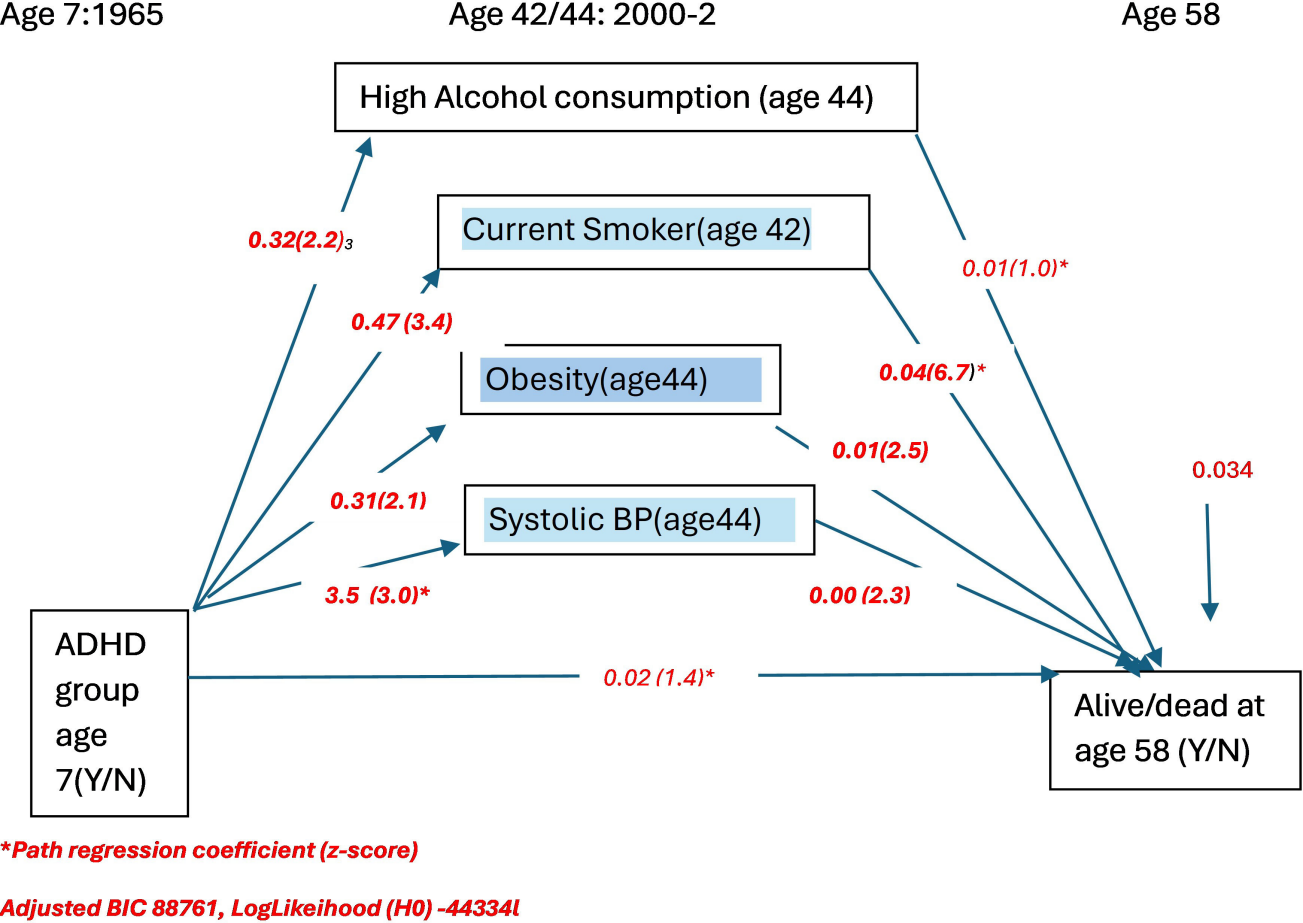
Path analysis of relationships between ADHD and mortality incorporating individual cardiovascular risk factor mediators (Measure of obesity is BMI-derived obesity) (n=8016): path regression coefficients and z-scores.

These were tested using path analysis (see **Figure 1**) with ADHD group as exposure, mortality status as outcome, and cardiovascular risks as mediators. We included cardiovascular risk factors that were found to be associated with both ADHD and mortality status in the initial bivariate analysis. We compared models using different measures of obesity. As cardiovascular risk mediators were assessed at age 44 years, for path analyses, we had to restrict our outcome of premature death to those that had occurred after this age (i.e., between 44 and 58 years of age). These analyses were conducted using MPLUS version 8.1.

#### Sensitivity testing

As not all individuals who completed the age 7 ADHD assessment completed the age 44 biomedical assessment, we compared the mortality rate that we found to the mortality rate for the total sample (i.e., including all those who completed the ADHD assessment, whether or not they completed the biomedical assessment). We also examined the mortality rates for those individuals who died before age 44 to assess for bias because these individuals could not be included in the path analysis (as mediators were assessed after death).

#### Accounting for missing data

The main analyses for the paper are based on individuals who completed the age 7 assessments for ADHD as well as the age 44 biomedical assessment. Missing data analysis was carried out using multiple imputation with chained equations in STATA 16.1, using the set of auxiliary variables suggested by the guide to missing data in NCDS (19) as well as earlier measurements of midlife cardiovascular risk factors. The results from the pooled analyses of imputed data were compared to those from unimputed data to check for accuracy and reduce the chance of bias in the results.

## Results

### Sample description

At age 7, 14,184 individuals completed the ADHD assessment questionnaire. We identified 494 individuals (3.5%) who met our criteria for likely ADHD, of whom 335 (67.8%) were male and 159 (32.2%) were female.

There were 1,919 total deaths with a recorded date in the dataset, with just over one-third (approximately 688) estimated to have died at age 44 years or older. Many of these deaths occurred in individuals on whom there was no information at age 7 from either the parent or the teacher for the ADHD questions or had not completed the age 44 biomedical assessment.

For the whole sample, 951 (6.7%) individuals who completed the ADHD assessment at age 7 had died by age 58.

For the analysis in this paper, the sample was restricted to 8,016 individuals who also completed the age 44 biomedical assessment. In this sample, 239 were in the ADHD group (3.1% of total), and 7,777 were classified as not having ADHD. Of those with ADHD, most were male (63.2%: 151/239).

#### Bivariate associations

1. ADHD and mortality: For those individuals who completed both the age 7 ADHD assessment and the age 44 biomedical survey when mediators were assessed, two hundred and eighty-six (3.6%) of these 8,016 individuals died between the ages of 44 and 58 years. Of the deaths, 15 had occurred in the ADHD group (6.3% of the ADHD group) and 271 in the non-ADHD group (3.5% of the total in the non-ADHD group). The odds ratio (OR) for deaths in the ADHD group versus the non-ADHD group was 1.86 (95% CI 1.08–3.17).

2. Association between ADHD and individual cardiovascular risk factors: ADHD was associated with the following individual cardiovascular risk factors: BMI (*B* = 0.92; 95% CI 0.27–1.56), BMI-defined obesity (odds Ratio (OR) 1.36; 95% CI 1.02–1.81), waist–hip ratio (*B* = 0.03; 95% CI 0.02–0.04), hazardous alcohol consumption at age 44 years (OR 1.38; 95% CI 1.03–1.85), SBP (*B* = 3.50; 95% CI 1.36–5.63), DBP (*B* = 2.22; 95% CI 0.82–3.61), current cigarette smoking (OR 1.60; 95% CI 1.22–2.10), and TG levels (*B* = 0.24; 95% CI 0.02–0.46). The descriptive results are detailed in **Table 1**.

**Table 1:** Descriptive details for individual cardiovascular risk factors comparing non-ADHD 173 and ADHD groups.

Outcome	Non-ADHD group			ADHD group		
Continuous variables	Mean(n)	SD	95%CI	Mean(n)	SD	95%CI
BMI (kg/m2)	27.3 (7639)	27.3	27.2-27.5	28.3(234)	28.3	27.5-29.0
WHR	0.87(7715)	0.09	0.867-0.870-	0.90 (238)	0.09	0.887-0.909
SBP (mmHg)	126.4(7661)	16.3	126.1-126.8	129.9(233)	17.4	127.7-132.2
DBP (mmHg)	78.7 (7661)	10.7	78.4-78.9	80.9(233)	10.7	79.5-82.3
TG levels	2.03(6475)	1.53	2.00- 2.07	2.27(202)	1.89	2.01-2.54
Categorical variables	Total n	(n)		otal n	%(n)	
Current smoker	7519	28.0 (2103)		232	38.4 (89)
Obese	7639	24.2 (1850)		234	30.3 (71)	
Hazardous drinking	7211	25.9(1870)		212	32.5 (69)	

3. Association between individual cardiovascular risk factors and mortality: The association between individual cardiovascular risk factors and mortality are reported in **Table 2**.

**Table 2:** Associations between individual cardiovascular risk factors (assessed at age 184 42/44) and mortality status by age 58 (n=8016).

Cardiovascular risk factor (continuous measures)	Odds ratio (OR)	95% CI for OR		P value
		Lower	Upper	
Systolic blood pressure(unadjusted)	1.01	1.00	1.02	<0.001
Diastolic blood pressure	1.01	1.00	1.02	0.07
Waist-hip ratio(corrected)	50.3	13.9	182.1	<0.001
LDL cholesterol	1.03	0.89	1.20	0.68
Triglycerides (unadjusted)	1.01	0.96	1.08	0.67
Cardiovascular risk factor (categorical measures)	Odds ratio (OR)	95% CI for OR		P value
		Lower	Upper	
Obesity (BMI≥30 kg/m2)	1.50	1.16	1.95	0.002
Current smoking status	2.62	2.05	3.34	<0.001
Hazardous alcohol consumption: AUDIT score ≥8	1.44	1.10	1.89	0.008

In summary, premature mortality was associated with BMI-defined obesity, waist–hip ratio, mean systolic blood pressure, current smoking status at 42 years of age, and hazardous alcohol consumption at age 44 years.

#### Identifying risk mediators

We selected mediators based on plausible mechanisms supported by both prior research findings of probable causal association with ADHD as well as influence on premature mortality. The mediators selected were (1) current smoker at age 42 years (Y/N), (2) hazardous alcohol consumption at age 44 years (audit score ≥8 categorized Y/N), (3) systolic blood pressure, and (4) obesity.

**Figure 2:**
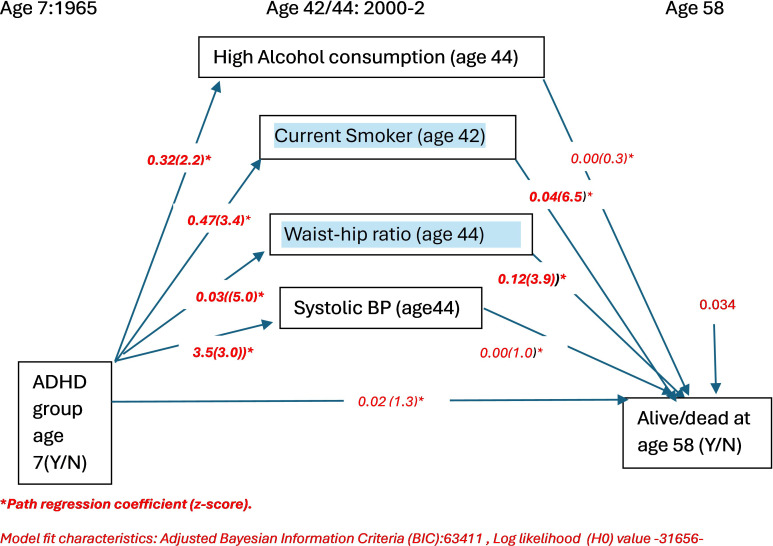
Association between ADHD and premature mortality including individual cardiovascular risk factor mediators with waist hip ratio as the index measure for obesity (n=8016): path regression coefficients and z-scores.

We tested two alternative models that varied in the measure of obesity used. **Figure 1** summarizes the results of the model that used BMI-based obesity. **Figure 2** summarizes the results of the model which used waist–hip ratio (WHR) as a marker of obesity. As can be seen, the model that included WHR as an index measure of obesity showed a superior fit (lower BIC and log likelihood) than that which used BMI-based obesity as the measure of obesity. Our results suggest that the relationship between ADHD and mortality appears to be mediated by current smoking at age 42 years and mid-life (age 44 years) waist–hip ratio (WHR).

If obesity based on BMI is used instead of WHR, then smoking, obesity, and mean systolic blood pressure mediated the relationship between ADHD and mortality (**Figure 1**).

### Sensitivity analyses

Mortality: For the 14,184 individuals who completed the age 7 ADHD assessment, not all completed the age 44 biomedical assessment. In this total sample, 951 individuals had died. Of the deaths, 51 (10.3%) had been within the ADHD group, and 900 (6.3%) had occurred in the non-ADHD group, with an OR (for deaths in the ADHD group versus the non-ADHD group) of 1.64 (95% CI 1.21–2.20).

We further checked the deaths that were recorded between the ages of 7 and 44 years. We observed that 339 individuals had died—319 in the non-ADHD group and 20 in the ADHD group. For this subgroup of earlier deaths, the OR for being in the ADHD group vs. non-ADHD group was 1.77 (95% CI 1.12–2.80).

### Missing data analysis

The results from the path analysis using imputed data are summarized in **Supplementary Figure 1**. The overall results of the path analysis with imputed data are generally consistent with the findings from the complete case analysis. However, some differences exist; the imputed data findings suggested the following: (1) an additional direct association between ADHD and premature death and (2) a negative association between hazardous drinking and premature death. There seems to be no plausible biological explanation for this latter finding, and findings from logistic regression on the imputed data do not show a negative association (**Supplementary Table 3**). Statistically, this situation can arise because of suppressor effects (20). Current smoking status may potentially be implicated, as there is now a much higher path coefficient (as compared to the complete case analysis) between current smoking and death. If current smoking status is removed from the path analysis, the direction of association between hazardous alcohol and death became as expected.

## Discussion

In a large UK-population-based cohort followed up to later middle age, we found that those with a history of childhood ADHD problems were at increased risk of dying by age 58 compared to those individuals who were not in our ADHD group. The risk was found to be mediated by specific modifiable cardiovascular risk factors that included smoking in mid-life and a high waist–hip ratio. If a high waist–hip ratio measurement was substituted by a BMI-based measure of obesity, then systolic blood pressure as well as smoking in mid-life and obesity mediated the link between ADHD status and premature mortality.

Current smoking has well-established links with premature mortality (21) and is an especially important risk factor to consider for those in mental health and neurodevelopmental services. A recent paper, using Mendelian randomization, highlighted that the reduced longevity associated with psychiatric disorders may be primarily driven by smoking (22). Individuals with ADHD are more likely to be smokers than the general population and may find it harder to stop smoking (23). However, in contrast to other neurodevelopmental conditions, ADHD symptoms can be reduced by medication as well as other interventions. Indeed ADHD medication appears to be one effective intervention to reduce smoking rates (24). The effectiveness of interventions for smoking cessation in those at higher risk for heavier smoking has been summarized (25). However, most smoking cessation programs for ADHD have not been shown to reduce the smoking rates. One multicenter randomized controlled trial aimed to optimize pharmacological treatment for ADHD as an adjunct to a smoking cessation program (behavioral counseling paired with the nicotine patch). The authors reported that while the smoking cessation rates between the treated (for ADHD) and placebo groups were similar, the number of cigarettes smoked dropped in the treated group (24). In a subsequent subgroup analysis, the smoking cessation rates in the ADHD treatment group were higher if the ADHD was more severe (26).

We also found that two alternative indices of obesity (waist–hip ratio (WHR) and BMI-derived obesity) mediated the relationship between ADHD and mortality. Several studies have found that ADHD is linked to markers of obesity—generally to obesity categorized by high BMI (27), but more recently the importance of the links between ADHD and elevated WHR have been highlighted (28). WHR has been found to be more closely linked to cardiovascular risk and mortality than BMI in several epidemiological studies (16, 29), and WHR is also more closely linked to vascular endothelial dysfunction (30). Interestingly, neurodevelopmental disorders (including ADHD) have also been linked to vascular endothelial growth factor (VEGF), which is closely linked to vascular endothelial function (31). The mechanisms of why ADHD is associated with obesity is not fully clear, but causal genetic links have been suggested by findings of studies using Mendelian randomization (MR) designs. One MR study also found a link between ADHD and coronary artery disease which was attenuated if obesity was controlled for (12). Another study which also used data from the North Finland Birth Cohort suggested that the links between ADHD and obesity had both a genetic and prenatal origin (32). ADHD may lead to obesity because of a dysfunctional brain reward system associated with overeating (33), and if ADHD and obesity co-occur, ADHD medication may reduce the level of obesity (34). Given that behavioral interventions in managing obesity need to be intensive and are only effective in a minority of patients (35) and that for individuals with ADHD obesity is closely linked to premature mortality, it may be argued that individuals with ADHD should be a priority group for both the effective treatment of ADHD itself and obesity prevention programs. If obesity occurs in those with ADHD, then it could be argued that those with ADHD should be prioritized for more effective obesity management strategies such as bariatric surgery (35) or anti-obesity medication (35).

Finally, we observed that mean systolic blood pressure was one of the mediators of the relationship between ADHD and premature mortality if BMI-defined obesity was used as the index of obesity rather than WHR. Previous research has found that childhood ADHD problems are associated with an increase in mean systolic blood pressure (36), and mean systolic blood pressure has been associated with increased all-cause mortality, but only for those above the 70th centile of SBP for their age and sex (37). However, our study did not examine clinically defined hypertension.

All of the mediators that we identified as potentially explaining the link between ADHD problems and premature mortality (cigarette smoking, obesity, and systolic blood pressure) are potentially modifiable. That is reassuring because our findings suggest that it is not ADHD itself that leads to premature mortality but rather associated physical health risks that are modifiable. While we selected established cardiovascular risk factors, many of these risks extend to other physical health conditions that can lead to premature mortality, including cancer. Although the risk factors that we identified are potentially modifiable, there are barriers to managing these risks. One key issue is whether people with a history of ADHD present to services or can access healthcare for both the assessment and management of ADHD and their physical health. A second barrier is the divide between services that assess and manage neurodevelopmental conditions such as ADHD—typically psychiatry—and those that manage cardiovascular risks that would be seen as the domain of primary care, not psychiatry. These divides between psychiatry/physical health and secondary/primary care are barriers to optimizing the physical health of those with ADHD. One way forward could be incentivized annual health checks conducted in primary care for those with ADHD, but different service models require evaluation.

### Limitations

Although this study has strengths, including a large population-based cohort that has been followed up to age 58 and that included mental health/neurodevelopmental and physical health measures, there are a number of limitations.

First, like nearly all population cohorts, once NCDS participants reached adult life, ADHD was no longer assessed. However, ADHD shows a high level of persistence across adult life.

Second, the ADHD measure was questionnaire-based. However, the prevalence rate generated using two informants in line with the population prevalence of ADHD and a broader definition in childhood mean that we are likely to have underestimated the rate of premature mortality.

Third, in the path analysis, we were restricted to including deaths that occurred after the biomedical assessment at age 44 years (cardiovascular risk factors) up to age 58 years. This meant that the path analyses only included a relatively small number of deaths. However, similar odds ratios of premature deaths in the complete case analysis to that for the larger sample (i.e., including deaths even in those who did not complete the biomedical assessment) suggest that these are similar.

## Conclusion

In a population-based cohort followed to later mid-life, we found that individuals with childhood ADHD problems had a higher premature mortality rate than individuals without ADHD. The association between ADHD and mortality was mediated by current smoking status at age 42 years and either waist–hip ratio or both BMI-derived obesity and mean systolic blood pressure at age 44 years. Our findings suggest that aggressive management of mid-life smoking and obesity should be a priority to reduce premature mortality in those with ADHD.

## Data Availability

The original contributions presented in the study are included in the article/**Supplementary Material**. Further inquiries can be directed to the corresponding author.
