# Supplementary material for: Association between childhood ADHD problems and premature mortality: identifying modifiable cardiovascular mechanisms in a UK population cohort
Source: Front Psychiatry. 2026 May 11;17:1764335. doi: 10.3389/fpsyt.2026.1764335 (PMC13199278; doi:10.3389/fpsyt.2026.1764335)
Supplement: Supplementary file 1 [file Table1.docx]

**Supplementary Materials: Association between childhood ADHD problems and premature mortality: identifying modifiable cardiovascular mechanisms in a UK population cohort**

**Summary of multiple imputation results**

Variables used in the imputation (variable names in brackets) ;

**Imputed variables** (at age 44-45 Biomedical assessment unless otherwise specified) :

Hazardous alcohol consumption (Audit_score_HIGH), Current smoker -at age 42(Currentsmoker), Triglycerides(trig), Systolic blood pressure (SBP_meannew), Body Mass index (bmi_biomedical), Low density lipoprotein ( LDL2), Waist Hip ratio (WHR_recoded,

**Predictor variables**:

***At birth****:* Sex (gender) interaction term ADHD and sex (adhdXgender) Number of persons per room-birth(aoconn512_trimmed_ord_new), Social class of mother’s father when she left school (acatnn660_collapsed_ord), Social class of mother’s husband (acatnn236_collapsed_ord) ***At age 7*** Dad stayed on at school after minimum age( bbinnn194_cat) BMI(BMI7_ rounded), Attendance (bcatnn458_ord) Cognitive ability summary (cogabilat7_new), Social problems -alcoholism etc. (socialdiffscore)

***At age 11****:* Child’s positive activities outside school (cconnage11dv32), BMI (BMI_11)

***At age 16*** Emotional or behavioural problem (dbinnn2021_new_cat), How long since child drank alcohol (dcatnn2888) Test 2 – mathematics comprehension (dconnn2930) Conduct problems (Ext16MTDec18_trimmed) Sum of good activities performed outside school (dconnage16dv47), BMI (BMI16_rounded

Death by age 58

**Supplementary Table 1: Descriptive details for missing data for individual cardiovascular risk factors (percentage of missing data for 14185 individuals who completed an ADHD assessment at age 7)**

| Variable | Missing | Total | Percent Missing |
| --- | --- | --- | --- |
| Systolic blood pressure | 6,293 | 14,185 | 44.36 |
| Triglyceride levels | 7,510 | 14,185 | 52.94 |
| Hazardous alcohol consumption | 6,764 | 14,185 | 47.68 |
| Current smoker | 6,436 | 14,185 | 45.37 |
| Low density lipoprotein | 7,856 | 14,185 | 55.38 |
| Waist-hip ratio | 6,172 | 14,185 | 43.51 |

Multiple imputation was carried out in Stata using chained equations.

**Code was : mi impute chained (logit) Audit_score_HIGH Currentsmoker (regress) trig SBP_meannew bmi_biomedical LDL2 WHR_recoded** **cconnage11dv32 dconnage16dv47= gender adhdXgender** **BMI7_ rounded BMI_11 socialdiffscore** **dcatnn2888 bbinnn194_cat** **Ext16MTDec18_trimmed BMI16_rounded dconnn2930 dbinnn2021_new_cat** **bcatnn458_ord aoconn512_trimmed_ord_new acatnn236_collapsed_ord** **acatnn660_collapsed_ord dbinnn2021_new_cat cogabilat7_new deaths_post_1965, add(10) rseed (53421) force savetrace(trace1,replace)**

**Supplementary Figure 1: Post-imputation Path Analysis of relationships between ADHD and mortality incorporating individual cardiovascular risk factor mediators (Waist hip ratio (WHR) as measure of obesity) (n=119074)**

Age 7:1965 Age 42/44: 2000-2 Age 58

High Alcohol consumption (age 44)

Current Smoker(age 42)

***0.07***

***-0.01(-3.95)***

***0.40(7.4****)_3_*

***034 (6.7)***

WHR Ratiobesity(age44)

***0.13(20.0) (6.7)***

***0.15 (5.22)***

***003 (16.1)***

Systolic BP(age44)

***0.00 (0.59)***

***3.26 (8.41)***

ADHD group age 7(Y/N)

Alive/dead at age 58 (Y/N)

***0.016 (3.5)***

***Adjusted BIC 4980000 LogLikeihood (H0 ) -248927***

**Supplementary Table2: Logistic regression: ADHD, systolic blood pressure (SBP), hazardous alcohol consumption (High Alcohol), low density lipoprotein (LDL), Waist hip ratio (WHR) and current smoking status as predictors of premature mortality using imputed data (n=35,373)**

| **Predictors** | **Odds Ratio (OR)** | **Standard error** | **Z** | **P test** | **95% Confidence levels for OR** | |
| --- | --- | --- | --- | --- | --- | --- |
|  |  |  |  |  | Lower | Upper |
| SBP | 1.00 | 0.00(1) | -2.63 | 0.008 | 0.99 | 1.0 |
| WHR | 3.00 | 1.01 | 3.24 | 0.001 | 1.54 | 5.81 |
| High alcohol | 1.20 | 0.07 | 3.21 | 0.001 | 1.07 | 1.34 |
| ADHD group | 1.28 | 0.17 | 1.94 | 0.053 | 1.0 | 1.65 |
| LDL | 1.02 | 0.03 | 0.80 | 0.422 | 0.96 | 1.08 |
| Current smoker | 2.98 | 0.16 | 20.85 | 0.000 | 2.69 | 3.30 |
|  |  |  |  |  |  |  |

**Outcome Premature mortality (deaths age 7-58)**

Log likelihood (LL) = -6299.9896 LR chi2(6) = 512.7

A similar pattern of results was found if BMI-based obesity was used as a measure of obesity instead of WHR(n=29,949). However, model fit slightly worse: LL= -4527.8 and LR chi2(6)= 420.3.

**Supplementary Table3: Logistic regression: ADHD, systolic blood pressure (SBP), hazardous alcohol consumption (High Alcohol), low density lipoprotein (LDL), Waist hip ratio (WHR) and current smoking status as predictors of premature mortality using complete case analysis (n=5653)**

| **Predictors** | **Odds Ratio (OR)** | **Standard error** | **Z** | **P test** | **95% Confidence levels for OR** | |
| --- | --- | --- | --- | --- | --- | --- |
|  |  |  |  |  | Lower | Upper |
| SBP | 1.00 | 0.01 | 0.63 | 0.53 | 0.99 | 1.01 |
| WHR | 16.70 | 17.12 | 2.74 | 0.006 | 2.24 | 124.6 |
| High alcohol | 1.10 | 0.19 | 0.55 | 0.58 | 0.78 | 1.54 |
| ADHD group | 1.77 | 0.61 | 1.67 | 0.10 | 0.91 | 3.47 |
| LDL | 0.91 | 0.08 | -1.10 | 0.27 | 0.77 | 1.08 |
| Current smoker | 2.51 | 0.40 | 5.81 | 0.000 | 1.84 | 3.42 |
|  |  |  |  |  |  |  |

LL -747.5 LR Chi2(6)=52.0
